# High‐Dimensional Immune Profiling Identifies Circulating NKT‐Like Cells Associated With Severity Outcome in Acute Pancreatitis at Disease Onset

**DOI:** 10.1111/imm.70143

**Published:** 2026-05-12

**Authors:** Carolina González de Castro, Mª. Lourdes Ruiz Rebollo, Paloma Cal‐Sabater, Elisa Arribas‐Rodríguez, Álvaro Martín‐Muñoz, Alejandro Gonzalez del Hierro, Marina Perez Mazzali, Jessica Matesanz‐Isabel, Hugo Gonzalo‐Benito, Sara Cuesta‐Sancho, David Bernardo

**Affiliations:** ^1^ Mucosal Immunology Laboratory Institute of Biomedicine and Molecular Genetics (IBGM), Universidad de Valladolid‐CSIC Valladolid Spain; ^2^ Gastroenterology Department Hospital Clínico Universitario de Valladolid Valladolid Spain; ^3^ Flow Cytometry Unit Institute of Biomedicine and Molecular Genetics (IBGM), Universidad de Valladolid‐CSIC Valladolid Spain; ^4^ Unidad de apoyo a la investigación, Hospital Clínico Universitario Valladolid Spain; ^5^ Instituto de Investigación Biosanitaria de Valladolid (IBioVALL) Valladolid Spain; ^6^ Centro de Investigaciones Biomedicas en Red de Enfermedades Infecciosas (CIBERINFEC) Madrid Spain

**Keywords:** acute pancreatitis, biomarkers, immune system, NKT‐like cells, spectral cytometry

## Abstract

Acute pancreatitis (AP) patients can be classified into mild or severe outcome based on the presence of organ failure days during hospitalization. However, there are no biomarkers that can predict severity outcome. This study aim is to perform an unbiased characterization of the circulating immunome of AP patients at hospital admission aiming to identify novel biomarkers which could predict disease outcome prior to the onset of organ damage and clinical deterioration. Peripheral blood mononuclear cells (PBMC) were collected from newly hospitalized AP patients. Based on their evolution, patients were classified following the Modified Atlanta classification into mild acute pancreatitis (mAP) (*n* = 20) as well as moderately and/or severe acute pancreatitis (ms‐sAP) (*n* = 20). PBMC were analysed by high‐dimensional spectral cytometry with unsupervised dimensionality reduction and clustering algorithms based on the subsequent outcome of the patients. Results were further validated by classical gating and functional approaches with a smaller cohort (*n* = 8 mAP and *n* = 8 ms‐sAP). A total of 120 different immune cell clusters were identified and characterized in AP patients at hospitalization. Following computational analysis and classical gating validation approaches, circulating CD2^+^CD8^dim^ NKT‐Like cells were found to be specifically reduced in ms‐sAP patients. Moreover, NKT‐Like cell subsets from these patients displayed an IL‐15 induced expansion of NKG2a and NKp30 referred to mAP. Patients with AP at hospitalization with subsequent ms‐sAP display a specific reduction of NKT‐Like cells coupled with an expanded function. Hence, quantifying the phenotype and/or function of circulating NKT‐Like cells during hospital admission could serve as a predictive biomarker for AP severity, aiding in early stratification and personalized treatment strategies.

## Graphical abstract

High‐dimensional spectral cytometry of peripheral blood mononuclear cells at hospital admission revealed that acute pancreatitis patients who subsequently developed moderately‐to‐severe disease exhibited a specific reduction of circulating CD2 + CD8dim NKT‐like cells, which appear to acquire an altered cytotoxic‐like phenotype upon IL‐15 stimulation. Quantifying the phenotype and function of circulating NKT‐like cells at admission could therefore serve as an early predictive biomarker of acute pancreatitis severity, enabling risk stratification and personalized treatment.

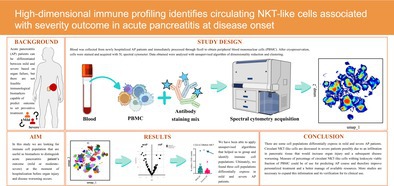

**Abbreviations**
AP: acute pancreatitis
FMO: fluorescence minus one
HC: healthy control
mAP: mild acute pancreatitis
ms‐sAP: moderately severe–severe acute pancreatitis
PBMC: peripheral blood mononuclear cells
UMAP: uniform manifold approximation and projection

## 1 Introduction

Acute pancreatitis (AP) is one of the most common gastroenterology hospital admissions in the Western world, with a steadily increasing incidence over the last decades [1, 2]. AP is an inflammatory and heterogeneous disease with most patients experiencing a mild course; however, approximately one‐third of AP patients may develop local or systemic complications and organ failure, which significantly increased morbidity and mortality [3]. Therefore, early classification of severity in AP is crucial to identify patients at higher risk of poor outcomes, enabling closer monitoring, targeted treatments and appropriate allocation to intensive care units.

Several scoring systems have been developed for this purpose, yet they have demonstrated only moderate accuracy in predicting severity in AP [4]. Therefore, novel approaches are urgently needed to better predict the disease course in this disease. Immune system plays a critical role in determining the severity of AP, as well as in the development of local and systemic complications and organ failure [5]. Evidence from both experimental models and clinical studies highlights the interplay between pancreatic acinar cells and immune system. Lui et al. [6] identified 44 immune cell traits associated with AP and 36 traits unidirectionally associated with sepsis. In animal models, Demols et al. [7] found CD4^+^ T‐cells to play a key role in the development of tissue injury in mice AP. Moreover, Glaubitz et al. [8] observed an overall activation of T‐cells in relation with AP severity and described that T‐cells depletion in immuno‐competent mice lowered disease severity. In human research, testing immune markers in the inflammatory pathways of AP, has also been performed. Indeed, increased circulating levels of CD4^+^CD25^+^CD127^high^ T‐cells in the early phase of AP correlates with subsequent organ failure in AP was found [9] while Shi et al. [10] also found that lower CD4^+^ T‐cell levels related to organ failure in AP of any aetiology. Conversely, the research by Minkov et al. [11] describes that AP patients who developed a severe episode in the course of the disease had elevated percentage of CD4^+^CD25^+^CD127^low/neg^ cells early on admission. In a similar manner, Pietruczuk et al. and Qui et al. [12, 13] described a significant depletion of peripheral blood T‐ and B‐cells in early stages of the disease, mostly in patients who develop severe episodes. Nevertheless, all these results, remain still insufficient to clearly define the precise role of the various lymphocyte subsets in human AP and their contribution to the severity of this life‐threatening condition.

## 2 Aim

Hence, we hereby aimed to fulfil this gap in the knowledge of the natural course of AP. To that end, we performed an unbiased characterization of the circulating immunome by means of spectral cytometry and machine learning‐based data analysis from AP patients during the early stage of the disease (within the first 24 h of hospitalization because of the onset of abdominal pain). This study provides therefore a comprehensive and in‐depth analysis of the immune system from these patients, with the purpose of identifying novel biomarkers capable of predicting disease severity at the early stage of the disease.

## 3 Methods

### 3.1 Study Subjects

Patients diagnosed with AP and admitted to our unit were enrolled in a prospective database, in which clinical, analytical, radiological and demographic data as well as serum samples are collected following the signing of informed consent.

The diagnosis of AP was carried out according to the revised Atlanta Classification [14] which requires two of the three following criteria: (1) acute onset of severe epigastric pain, (2) serum amylase or lipase activity at least three times greater than the upper limit of normal, (3) characteristic findings of AP on radiology. The exclusion criteria were: (1) Patients who did not accept the inclusion in our database or did not sign the informed consent. (2) Patient admission more than 5 days after the onset of pain or patients referred from other hospitals. (3) Patients who could not be followed up.

Data recorded included age, sex, abdominal perimeter, body mass index (BMI) and smoking and alcoholic status on admission. The aetiology of the episode of AP was attributed to one of the following: gallstones, alcohol, post‐endoscopic retrograde cholangiopancreatography, idiopathic and others. The presence of single or multiple organ failure (respiratory, cardiovascular and renal) was registered according to the modified Marshall score system and was defined as a score of 2 or more for one of those three organs. Local complications included pancreatic or peripancreatic collections. The severity of AP episodes was classified following the Modified Atlanta classification into mild acute pancreatitis (mAP) (no organ failure and no local or systemic complications), moderately severe pancreatitis (organ failure that resolves within 48 h and/or local or systemic complications) and severe acute pancreatitis (ms‐sAP) (single or multiple organ failure which persists more than 48 h). The recruitment selection for our study included, as a first discovery cohort, 20 patients suffering from mAP and 20 patients with a moderately severe–severe AP episode (ms‐sAP) (Table 1). Additionally, 20 age and sex–matched healthy individuals were enrolled as healthy controls (HCs), with median age and IQR 70 (37–94) and 14 (70%) male HC. None of the participants‐patients or controls had any immunological, autoimmune or oncological disease which conditions that could potentially affect their immune profile. In addition, a second cohort of 16 patients, 8 mAP and 8 ms‐sAP, was recruited for functional validation (Table 2).

**TABLE 1:** Demographic, analytic and clinic characteristics of patients studied in discovery cohort. Description of demographic variables of three groups of patients used in cohort 1 of this study.

		Mild acute pancreatitis (mAP)	Severe acute pancreatitis (sAP)
*n*		20	20
Age		73 (41–100)	73 (42–96)
Gender	Female	7 (35%)	7 (35%)
	Male	13 (65%)	13 (65%)
Body mass index	< 25	5 (25%)	5 (25%)
	25–30	10 (50%)	8 (40%)
	> 30	5 (25%)	7 (35%)
Abdominal perimeter (cm) median/IQ range		105 (90.8–108.3)	105 (95–111.3)
Alcohol status	No	16 (80%)	14 (70%)
	Yes	4 (20%)	5 (25%)
	Former (> 6 months)	0 (0%)	1 (5%)
Smoking status	No	11 (55%)	10 (50%)
	Yes	3 (15%)	4 (20%)
	Former (> 6 months)	6 (30%)	6 (30%)
Aetiology	Gallstone related	14 (70%)	13 (65%)
	Alcohol	0 (0%)	2 (10%)
	Post ERCP	1 (5%)	1 (5%)
	Others	0 (0%)	0 (0%)
	Idiopathic	5 (25%)	4 (20%)
High blood pressure	No	10 (50%)	8 (40%)
	Yes	10 (50%)	12 (60%)
Diabetes mellitus	No	17 (85%)	15 (75%)
	Yes	3 (15%)	5 (25%)
Albumin (mg/dL) median/IQ range		3.8 (3.6–4.1)	3.8 (3.2–4)
Triglycerides (mg/dL) median/IQ range		90.5 (78–120.8)	69.5 (61.5–88.3)
BUN (mg/dL) median/IQ range		19.4 (15.3–26.5)	30.8 (18.2–37.6)
Leukocytes ×10^3^/μL median/IQ range		9990 (8660–14827.5)	14245 (11752.5–16452.5)
CRP (mg/dL) median/IQ range		4 (2–13)	12.5 (4.8–33.5)
Creatinine (mg/dL) median/IQ range		1 (0.9–1.3)	1.2 (0.8–1.6)
Hematocrito (mg/dL) median/IQ range		42.2 (39.1–46.3)	42.9 (37.1–46.4)
Abdominal pain (hours) median/range		12 (7.5–24)	12 (8–24)

**TABLE 2:** Demographic, analytic and clinic characteristics of patients studied in functional validation cohort. Description of demographic variables of two groups of patients used in cohort 2 of this study.

		Mild acute pancreatitis (mAP)	Severe acute pancreatitis (sAP)
*n*		8	8
Age		67 (42–84)	71 (47–84)
Gender	Female	6 (75%)	5 (62.5%)
	Male	2 (25%)	3 (37.5%)
Body Mass Index	< 25	4 (50%)	2 (25%)
	25–30	3 (37.5%)	4 (50%)
	> 30	1 (12.5%)	2 (25%)
Abdominal perimeter (cm) median/IQ range		97 (87.3–107.8)	102.5 (99–114.8)
Alcohol status	No	6 (75%)	7 (87.5%)
	Yes	2 (25%)	1 (12.5%)
	Former (> 6 months)	0 (0%)	0 (0%)
Smoking status	No	8 (100%)	5 (62.5%)
	Yes	0 (0%)	2 (25%)
	Former (> 6 months)	0 (0%)	1 (12.5%)
Aetiology	Gallstone related	5 (62.5%)	4 (50%)
	Alcohol	1 (12.5%)	1 (12.5%)
	Post ERCP	2 (25%)	1 (12.5%)
	Others	0 (0%)	0 (0%)
	Idiopathic	0 (0%)	2 (25%)
High Blood pressure	No	4 (50%)	2 (25%)
	Yes	4 (50%)	6 (75%)
Diabetes mellitus	No	5 (62.5%)	6 (75%)
	Yes	2 (25%)	2 (25%)
Albumin (mg/dL) median/IQ range		3.7 (3.7–3.8)	3.2 (3.2–4.1)
Triglycerides (mg/dL) median/IQ range		114 (92.3–139.5)	89.5 (69.8–118.8)
BUN (mg/dL) median/IQ range		18 (15.5–23.7)	25.8 (20.4–28.6)
Leukocytes ×10^3^/μL median/IQ range		10755 (8427.5–13107.5)	15860 (13660–18502.5)
CRP (mg/dL) median/IQ range		7.5 (3.8–22.5)	9 (3.3–59.7)
Creatinine (mg/dL) median/IQ range		0.8 (0.7–0.9)	1 (0.8–1.3)
Hematocrito (mg/dL) median/IQ range		37.2 (36.5–42)	46.5 (44.5–47.7)
Abdominal pain (hours) median/range		24 (20–30)	10 (7.5–15)

Due to the limited number of patients with severe outcomes (*n* = 3 in discovery cohort and *n* = 2 in functional cohort), mild–severe and severe patients were combined in all analyses throughout this study. This limitation should be considered when interpreting the findings.

### 3.2 Blood Sampling and Laboratory Procedure

Blood samples were obtained in EDTA tubes for both cohorts, within 24 h after hospitalization and at a median of 12 h (7.5–24) from the onset of abdominal pain, between March 2022 and September 2024. Total peripheral blood mononuclear cells (PBMC) were enriched following centrifugation over a ficoll gradient (Cytiva Ficoll‐PaqueTM PLUS). PBMC were further cryopreserved (90% foetal bovine serum [FBS] + 10% dimethyl sulphoxide [DMSO]) in N_2_ until used.

### 3.3 Antibody Staining and Spectral Cytometry Acquisition

For the descriptive study, a total of 2 million PBMC were thawed from both controls, and AP patients at hospital admission. Once washed, cells with stained with monoclonal antibodies and subsequently characterized by spectral cytometry (CyTek Aurora 5‐laser) according to the OMIP‐069 panel and protocol [15] with minor modifications on the panel as shown in Table S1. Briefly, the Live/Dead Fixable Blue Dead Cell Stain Kit (Molecular Probes, Thermo Fisher Scientific) was added to exclude dead cells from the analysis. Brilliant Stain Buffer and True‐Stain Monocyte Blocker were also added prior to staining with the antibodies to obtain optimal fluorescence of the desired cells. PBMCs were washed with Wash buffer (500 mL phosphate‐buffered saline [PBS] + 1 g Bovine serum albumin [BSA] 0.2% + 0.445 g sodium azide [NaN_3_] 0.089%) and incubated in the dark at room temperature during the staining process. Finally, cells were fixed with 0.8% paraformaldehyde in Wash buffer in the dark for 10 min, washed with Wash buffer and stored at 4°C until acquired within 24 h in a 5‐laser spectral cytometer (Aurora, Cytek).

### 3.4 Cell Culture

Frozen cells from Cohort 2 were thawed at 37°C and then were cultured in 96‐well flat‐bottom (Thermofisher) with supplemented media (RPMI (Gibco) with 10% FBS (Gibco), 1% glutamine (Gibco), 1% Penicilin/streptomycin (Gibco) and 0.1% gentamicine) in a concentration of 200 000 PBMCs in 200 μL per well. Cells from each patient were cultured in resting conditions as well as in the presence 50 ng/mL of IL‐15 (BD Biosciences), for 18 h. Following culture, cells were harvested and stained as above with the panel described in Table S2. Cells were further fixed, preserved and acquired as previously explained.

### 3.5 Computational Cytometric Analysis

The OMIQ Data Science platform (Omiq Inc. 2022) was used following transformation of the data; the scale, parameters and cofactors were set as suggested by the platform. The implemented data analysis is the one that we have previously described and applied [16, 17, 18, 19], and it was applied for the discovery cohort. Briefly, the data‐cleaning PeacoQC algorithm was applied to remove outlier events in spectral cytometry data files due to abnormal flow behaviour resulting from clogs and other common technical problems. Subsequently, a manual discard was performed to eliminate cell debris and doublets and to select viable leucocytes (CD45^+^ cells), which were used for subsequent analysis.

The Uniform Manifold Approximation and Projection (UMAP) algorithm was used for the exploratory analysis following a prior random subsampling to render a total of 4 000 000, making sure ensure that each cohort was equally represented. We next applied a FlowSOM algorithm to find similar cell metaclusters and separate them into groups in an unsupervised manner. The visual representation of both algorithms allows further subdivision of these metaclusters into clusters that provide a more accurate representation of all the phenotypic and functional subsets of the human immunome. A clustered heatmap was created using the clusters obtained in the previous point to represent the expression level of each phenotypic in each cluster. Dendrograms further grouped clusters and phenotypic markers associated by similarity (distance). Of note, patients with less than 35 000 events recorded were excluded from the unsupervised analysis, rendering, for the unsupervised analysis, 20 HC, 19 mAP and 18 ms‐sAP.

For the characterization of the phenotype and function of the cells from the functional validation cohort, a hierarchical approach within aimed immune subsets was performed.

In all cases, expression from fluorescence minus one (FMO) was used to define each marker expression.

### 3.6 Statistical Analyses

For the computational cytometry data, Volcano plots were constructed with the edgeR algorithm comparing cluster differences, a *p*‐value under 0.05 was considered statistically significant without further correction. Once the clusters showing significant differences were identified, a validation of the data was performed by classical hierarchical analysis. Using a modified gating strategy of OMIP‐69 panel, the percentages within the total viable leukocytes fraction (CD45^+^) of those clusters that stood out in the previous analysis were obtained and further analysed using GraphPad Prism9. Quantitative variables were expressed as mean and standard deviation, as they followed a normal distribution, and the parametric *t*‐student test was used. One‐Way ANOVA, Fisher test and *t*‐test comparisons were also applied as detailed in the Figure Legends. In all cases, a *p*‐value under 0.05 was considered statistically significant.

Generative artificial intelligence has been used for orthographical correction, translation and style enhancement in some paragraphs. All content has been critically reviewed and validated by the author to ensure accuracy and scientific relevance.

## 4 Results

### 4.1 High‐Dimensional Mapping of the Circulating Immunome in AP Reveals a Total of 120 Different Immune Cell Clusters

**FIGURE 1:**
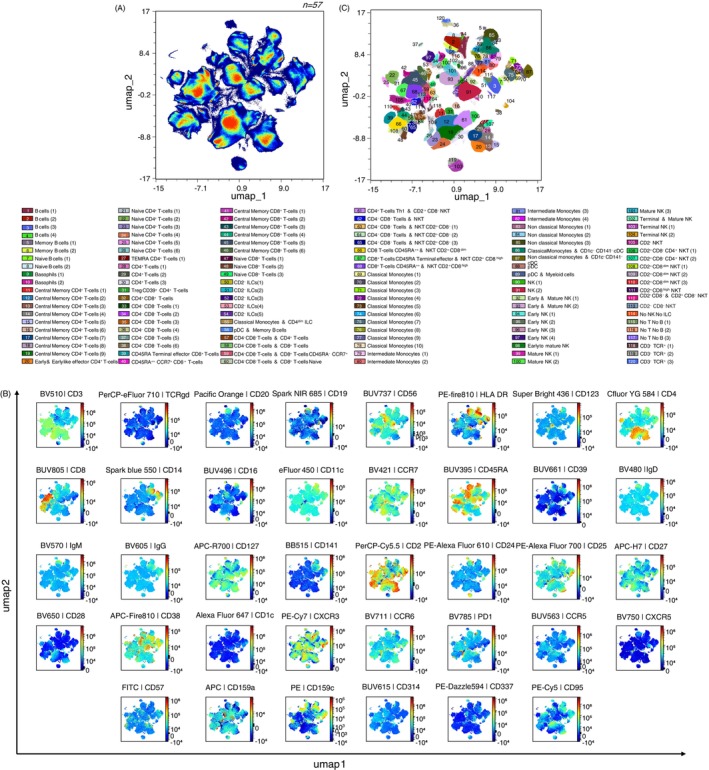
Unbiased characterization of the circulating immunome. (A) UMAP analyses was performed within total singlet viable CD45+ cells from samples with more than 35.000 events (*n* = 57). Subsequent down sampling to a total of 4 million events was performed being each cohort equally represented. Red represents higher number of grouped cells and gradient through yellow to blue lower number of cells. (B) Surface expression intensities for all remaining 38 analysed markers is shown. Colour code is based on the intensity where red represent higher expression and blue lower expression. (C) All 120 identified clusters are shown overlaid on the UMAP plot previously obtained. Each identified cluster is tagged by a specific colour and number as shown in the legend.

In order to visualize the circulating immune landscape from patients with AP, referred to controls, we first performed a UMAP analysis across all three cohorts (Figure 1A) showing in Figure 1B the relative expression of each marker. FlowSom analysis identified a total of 120 clusters as shown in (Figure 1C), with their specific phenotype being described in (Table 3 and Figure S1). Hence B cells (clusters number 1– 8), Monocytes (Clusters 69–85) and Myeloid antigen‐presenting cells (APC) (Clusters 86–89) were primarily located in the upper right region, while the central area was mainly composed of NK cells (Clusters 90–104) and some innate lymphoid cells (ILC) (Clusters 50–54). CD4^+^ T‐cells (Clusters 11–31) were located in the lower region and their cytotoxic CD8^+^ counterparts (Clusters 39–49) were found on the left side, while NKT‐like cells (Clusters 105–113) were distributed throughout these populations.

**TABLE 3:** Cell cluster identification for discovery cohort. For each of the 120 clusters identified by FlowSOM as shown in Figure 1C, their cell population and cell subset are shown, together with their phenotype and function.

Cluster	Population	Subset	Phenotypic expression	Functional expression
1	B cells	(1)	CD20, CD16, CCR7, CD45RA, CD127, IgD^+/−^, IgM_smere_, CD24_smere_, CD27_smere_	HLA‐DR, CD24, CXCR3, PD‐1, CXCR5
2	B cells	(2)	CD20, CD16, CCR7, CD45RA, CD127, IgD^+/−^, IgMsmere, CD24smere	HLA‐DR, CD24, CXCR3, PD‐1, CXCR5, NKG2c
3	B cells	(3)	CD20, CCR7, CD45RA, CD127, CD2, CD25, CD38, IgD^+/−^, IgM^+/−^, CD24_smere_, CD27_smere_	HLA‐DR, CXCR3, CXCR5, NKG2c
4	B cells	(4)	CD20, CD56, CD123, CD4, CCR7, CD45RA, CD39, IgD, IgM, CD24_smere_, CD25, CD27, CD28	HLA‐DR, CD24, CXCR3, CCR6, PD‐1, CXCR5, NKG2D
5	B cells	Memory (1)	CD20, CD4, CD16, CD11c, CD45RA, CD141	HLA‐DR, PD‐1, Fas, NKG2c
6	B cells	Memory (2)	CD20, CD16, CCR7, CD45RA, CD39, CD27_smere_	HLA‐DR, CXCR3, PD‐1, CXCR5^+/−^, Fas^+/−^, NKG2c
7	B cells	Naive (1)	CD20, CD19, CCR7, CD45RA, IgD, IgM, CD127, CD2, CD24_smere_, CD25, CD38^dim^	HLA‐DR, CXCR3_smere_, CCR6, CXCR5, NKG2c
8	B cells	Naive (2)	CD20, CD19, CCR7, CD45RA, CD39, IgD, IgM, CD24, CD25, CD38^dim^	HLA‐DR, CXCR3^+/−^, CCR6_smere_, PD‐1, CXCR5, NKG2c
9	Basophils	(1)	CD56, CD123, CD16, CCR7, CD45RA, CD39, IgM, CD25, CD28, CD38	CXCR3, CCR6, PD‐1, Fas, NKG2D, NKp30_smere_
10	Basophils	(2)	CD56, CD123, CD4, CCR7, CD45RA, CD39, IgM, CD127, CD2, CD25, CD28, CD38	CXCR3, CCR6, PD‐1, Fas, NKG2D, NKp30_smere_
11	CD4^+^ T‐cells	Central memory (1)	CD3, CD4, CD16, CCR7, CD127, CD2, CD25, CD27, CD28, CXCR3^+/−^	PD‐1, Fas, CCR5_smere_
12	CD4^+^ T‐cells	Central memory (2)	CD3, CD4, CD16, CCR7, CD127, CD2, CD25, CD27, CD28, CXCR3	PD‐1, Fas
13	CD4^+^ T‐cells	Central memory (3)	CD3, CD4, CCR7, CD127, CD2, CD25, CD27, CD28, CXCR3, CCR6_smere_	Fas, CXCR5^+/−^, NKG2c^+/−^
14	CD4^+^ T‐cells	Central memory (4)	CD3, CD4, CCR7, CD127, CD2, CD27, CD28, CD1c, CXCR3	Fas
15	CD4^+^ T‐cells	Central memory (5)	CD3, CD4, CCR7, CD127, CD2, CD25, CD27, CD28, CD1c_smere_, CXCR3, CCR6_smere_	HLA‐DR, PD‐1^+/−^
16	CD4^+^ T‐cells	Central memory (6)	CD3, CD4, CD16, CCR7, CD127, CD2, CD25, CD28, CXCR3	HLA‐DR, PD‐1, CCR5_smere_, Fas, NKG2c
17	CD4^+^ T‐cells	Central memory (7)	CD3, CD4, CCR7, CD127, CD27, CD28, CXCR3	
18	CD4^+^ T‐cells	Central memory (8)	CD3, CD4, CCR7, CD127, CD27, CD28_smere_, CXCR3	
19	CD4^+^ T‐cells	Central memory (9)	CD3, CD4, CD16, CCR7, CD127, CD27, CD28, CXCR3	Fas_smere_
20	CD4^+^ T‐cells	Early and early like effector	CD3, CD4, CCR7_smere_, CD127, CD2, CD25, CD27^+/−^, CD28, CXCR3^+/−^	CXCR5^+/−^, Fas
21	CD4^+^ T‐cells	Naive (1)	CD3, CD4, CCR7, CD45RA, CD127, CD27, CD28, CXCR3	
22	CD4^+^ T‐cells	Naive (2)	CD3, CD4, CCR7, CD45RA, CD127, CD2, CD27, CD28, CD38, CXCR3	
23	CD4^+^ T‐cells	Naive (3)	CD3, CD4, CD16, CCR7, CD45RA, CD127, CD2, CD27, CD28, CXCR3	PD‐1
24	CD4^+^ T‐cells	Naive (4)	CD3, CD4, CD16, CCR7, CD45RA, CD127, CD27, CD28, CXCR3	PD‐1
25	CD4^+^ T‐cells	Naive (5)	CD3, CD4, CCR7, CD45RA, CD127, CD2, CD27, CD28, CD38	
26	CD4^+^ T‐cells	Naive (6)	CD3, CD4, CD16, CCR7, CD45RA, CD127, CD2, CD27, CD28	PD‐1
27	CD4^+^ T‐cells	TEMRA	CD3, CD4, CD45RA, CD127, CD2, CXCR3	PD‐1_smere_, CD57_smere_, Fas_smere_, NKG2c_smere_
28	CD4^+^ T‐cells	Th1 (1)	CD3, CD4, CCR7^+/−^, CD127, CD2, CD27_smere_, CXCR3	PD‐1^+/−^, CCR5_smere_, CD57^+/−^, Fas, NKG2c
29	CD4^+^ T‐cells	Th1 (2)	CD3, CD4, CCR7_smere_, CD45RA^+/−^, CD127, CD2, CXCR3	PD‐1^+/−^, CD57_smere_, NKG2c^+/−^
30	CD4^+^ T‐cells	Th1 (3)	CD3, CD4, CD16, CCR7_smere_, CD127, CXCR3	
31	CD4^+^ T‐cells	Treg CD39^+^	CD3, CD4, CD16, CCR7_smere_, CD39^+/−^, CD127^+/−^, CD2, CD25, CD27^+/−^, CD28, CXCR3^+/−^	HLA‐DR^+/−^, PD‐1, CCR5_smere_, Fas
32	CD4^+^CD8^+^ T‐cells		CD3, CD4, CD8, CD16, CCR7, CD127, CD2, CD27_smere_, CD28	CXCR3^+/−^, PD‐1, Fas, NKG2D_smere_
33	CD4^−^CD8^−^ T‐cells	(1)	CD3, CCR7, CD127, CD27	CXCR3
34	CD4^−^CD8^−^ T‐cells	(2)	CD3, CCR7, CD127, CD27, CD1c	CXCR3
35	CD4^−^CD8^−^ T‐cells	(3)	CD3, CCR7_smere_, CD127, CD2, CD27, CD28_smere_	CXCR3, NKG2a^+/−^
36	CD4^−^CD8^−^ T‐cells	(4)	CD3, CD14, CD11c, CCR7_smere_, CD127, CD141, CD2, CD25, CD1c	CD24, CXCR3, CXCR5, Fas, NKG2c^+/−^, NKp30
37	CD4^−^CD8^−^ T‐cells	(5)	CD3, CCR7, CD45RA, CD127, CD2, CD27, CD28, CD38	HLA‐DR^+/−^, CXCR3, CXCR5, Fas, NKG2c^+/−^
38	CD4^−^CD8^−^ T‐cells	(6)	CD3, CD16, CCR7, CD45RA, CD127, CD27, CD28, CD38	HLA‐DR, CXCR3, PD‐1, CCR5_smere_, Fas, NKG2c^+/−^
39	CD8^+^ T‐cells	CD45RA Terminal effector	CD3, CD8, CD45RA^+/−^, CD127, CD2	CXCR3_smere_, PD‐1_smere_, CD57_smere_, Fas_smere_
40	CD8^+^ T‐cells	CD45RA++CCR7+	CD3, CD8, CD16, CCR7, CD45RA, CD127	CXCR3, NKG2c
41	CD8^+^ T‐cells	Central memory (1)	CD3, CD8, CCR7, CD127, CD27, CD28, CD1c	CXCR3
42	CD8^+^ T‐cells	Central memory (2)	CD3, CD8, CD16, CCR7^+/−^, CD127, CD2, CD27_smere_, CD28_smere_	HLA‐DR, CXCR3, PD‐1, CCR5, Fas, NKG2c, NKG2D
43	CD8^+^ T‐cells	Central memory (3)	CD3, CD8, CCR7_smere_, CD127, CD2, CD25, CD27_smere_, CD28_smere_, CD38	HLA‐DR, CXCR3, PD‐1^+/−^, CCR5_smere_, Fas, NKG2c
44	CD8^+^ T‐cells	Central memory (4)	CD3, CD8, CCR7_smere_, CD127, CD2, CD27, CD28^+/−^	CXCR3, Fas_smere_, NKG2c_smere_
45	CD8^+^ T‐cells	Central memory (5)	CD3, CD8, CD16, CCR7_smere_, CD45RA^+/−^, CD127, CD2, CD27^+/−^, CD28^+/−^	CXCR3, PD‐1, CCR5_smere_, CD57_smere_, Fas, NKG2D
46	CD8^+^ T‐cells	Central memory (6)	CD3, CD8, CD16, CCR7, CD127, CD2, CD27, CD28	CXCR3^+/−^, Fas, NKG2D
47	CD8^+^ T‐cells	Naive (1)	CD3, CD8, CCR7, CD45RA, CD127, CD27, CD1c	CXCR3
48	CD8^+^ T‐cells	Naive (2)	CD3, CD8, CCR7, CD45RA, CD127, CD2, CD27, CD28	CXCR3
49	CD8^+^ T‐cells	Naive (3)	CD3, CD8, CD16, CCR7, CD45RA, CD127, CD27, CD28	CXCR3, PD‐1, NKG2D
50	ILCs	CD2^−^ (1)	CCR7, CD127, CD27, CD38	CXCR3, NKG2c
51	ILCs	CD2^−^ (2)	CCR7, CD45RA, CD127, CD27, CD1c	HLA‐DR, CXCR3, CXCR5, NKG2c
52	ILCs	CD2^−^ (3)	CD16, CCR7, CD127, CD27, CD38	CXCR3
53	ILCs	CD2^−^ (4)	CD16, CCR7_smere_, CD45RA, CD127	CXCR3, NKG2c
54	ILCs	CD2^−^ (5)	CD16, CCR7, CD127	CXCR3, NKG2c^+/−^
55	Mixed No T‐cells	Classical monocytes and CD4^dim^ ILC	HLA‐DR_smere_, CD4, CD14, CD16_smere_, CD11c_smere_, CCR7, IgG, CD127, CD2, CD38	CXCR3, CD57, Fas
56	Mixed No T‐cells	pDC and memory B cells	HLA‐DR, CD123^+/−^, CD16, CCR7, CD45RA, CD127, CD20^+/−^, CD19^+/−^, CD38^+/−^	CXCR3, PD‐1, CXCR5, Fas^+/−^, NKG2c
57	Mixed Real T‐cells	CD4^−^CD8^−^ T‐cells and CD4^+^ T‐cells	CD3, CD4^+/−^, CCR7, CD45RA_smere_, CD127	CXCR3
58	Mixed Real T‐cells	CD4^−^CD8^−^ T‐cells and CD8^+^ T‐cells	CD3, CCR7, CD45RA^+/−^, CD127	CXCR3, NKG2c^+/−^
59	Mixed Real T‐cells	CD4^−^CD8^−^ T‐cells and CD8^+^ T‐cells CD45RA^−^CCR7^+^	CD3, CD8^+/−^, CD16, CCR7, CD45RA_smere_, CD127	CXCR3
60	Mixed Real T‐cells	CD4^−^CD8^−^ T‐cells and CD8^+^ T‐cells Naive	CD3, CD8^+/−^, CCR7^+/−^, CD45RA, CD127, CD2, CD27^+/−^	CXCR3, Fas^+/−^
61	Mixed T‐cells	CD4^+^ T‐cells Th1 and CD2^+^CD8^−^ NKT	CD3, CD56^+/−^, CD4, CD16, CD127, CD2	CXCR3, PD‐1^+/−^, CCR5_smere_, CD57^+/−^, Fas
62	Mixed T‐cells	CD4^−^CD8^−^ Tcells and NKT	CD3, CD56^+/−^, CD8^+/−^, CD16, CCR7, CD45RA, CD127, CD2_smere_	CXCR3, PD‐1, Fas, NKG2D
63	Mixed T‐cells	CD4^−^CD8^−^ Tcells and NKT CD2^+^CD8‐ (1)	CD3, CD56_smere_, CD16, CCR7, CD127, CD2, CD27_smere_, CD28_smere_	CXCR3, CCR5, Fas_smere_, NKG2D
64	Mixed T‐cells	CD4^−^CD8^−^ Tcells and NKT CD2^+^CD8^−^ (2)	CD3, CD56^+/−^, CD16, CCR7, CD45RA^+/−^, CD127, CD2	HLA‐DR, CXCR3, PD‐1, CCR5_smere_, Fas, NKG2c
65	Mixed T‐cells	CD4^−^CD8^−^ Tcells and NKT CD2^+^CD8^−^ (3)	CD3, CD56^+/−^, CD127, CD2, CD28_smere_	CXCR3, CCR5^+/−^, Fas_smere_, NKG2a^+/−^, NKG2c^+/−^
66	Mixed T‐cells	CD8^+^ T‐cells CD45RA^++^ and NKT CD2^+^CD8^dim^	CD3, CD56^+/−^, CD8, CD45RA, CD127, CD2, CD27_smere_	CXCR3, CD57_smere_, Fas_smere_, NKG2c_smere_
67	Mixed T‐cells	CD8^+^ T‐cells CD45RA terminal effector and NKT CD2^+^CD8^high^	CD3, CD56^+/−^, CD8, CD16, CD45RA, CD2	CXCR3, PD‐1, CCR5_smere_, CD57^+/−^, Fas, NKG2D
68	Mixed T‐cells	CD8^+^ T‐cells CD45RA^++^ and NKT CD2^+^CD8^high^	CD3, CD8, CD16, CD45RA, CD127, CD2	CXCR3, PD‐1, CCR5_smere_, CD57_smere_, Fas_smere_, NKG2D
69	Monocytes	Classical (1)	HLA‐DR, CD4, CD14, CD11c_smere_, CCR7, IgG, CD127, CD2, CD25, CD38	CXCR3, CCR6_smere_, CXCR5, CD57_smere_, Fas, NKG2c, NKp30_smere_
70	Monocytes	Classical (2)	HLA‐DR, CD4, CD14, CD16_smere_, CD11c, CD39, CD127, CD141, CD2, CD25, CD38, CD1c	CXCR3, CXCR5, CD57, Fas, NKG2c, NKp30_smere_
71	Monocytes	Classical (3)	HLA‐DR, CD4, CD14, CD16_smere_, CD11c_smere_, CD127, CD2, CD25, CD38	CD57, Fas
72	Monocytes	Classical (4)	HLA‐DR, CD4, CD14, CD16_smere_, CD11c_smere_, CD127, CD2, CD25, CD38	CD57, Fas, NKG2c
73	Monocytes	Classical (5)	HLA‐DR, CD4, CD14, CD16_smere_, CD11c_smere_, CCR7, CD127, CD2, CD25, CD38	CXCR3, CXCR5, CD57, Fas, NKG2c
74	Monocytes	Classical (6)	HLA‐DR, CD4, CD14, CD16_smere_, CD11c_smere_, CCR7, CD39, IgG, CD141, CD38	CXCR3, PD‐1, CCR5, CXCR5, Fas, NKG2c
75	Monocytes	Classical (7)	HLA‐DR, CD14, CD11c_smere_, CCR7, CD127, CD38	CXCR3, CXCR5, Fas, NKG2c
76	Monocytes	Classical (8)	HLA‐DR, CD14, CD16_smere_, CD11c_smere_, CCR7, CD39, CD127, CD141, CD38	CXCR3, PD‐1, CXCR5, Fas, NKG2c
77	Monocytes	Classical (9)	HLA‐DR, CD14, CD16_smere_, CD11c_smere_, CCR7, CD127, CD38	CXCR3, CXCR5, Fas
78	Monocytes	Classical (10)	HLA‐DR, CD4, CD14, CD16_smere_, CD11c, CCR7, CD39, CD141, CD38	CXCR3, CXCR5, Fas, NKG2c
79	Monocytes	Intermediate (1)	HLA‐DR, CD4, CD14, CD16, CD11c, CCR7, CD39, CD141, CD38	PD‐1, CCR5, CXCR5, Fas, NKG2c, NKp30
80	Monocytes	Intermediate (2)	HLA‐DR, CD14, CD16, CD11c_smere_, CCR7, CD39, IgG, CD141, CD38	CCR5, CXCR5, Fas, NKG2c^+/−^
81	Monocytes	Intermediate (3)	HLA‐DR, CD14, CD16, CD11c, CCR7, CD39, IgG, CD38	CXCR3, CXCR5, Fas, NKG2c
82	Monocytes	Intermediate (4)	HLA‐DR, CD4, CD14, CD16, CD11c, CD39, IgG, CD141, CD38	CXCR3, PD‐1, CCR5_smere_, CXCR5, Fas, NKG2c, NKp30_smere_
83	Monocytes	Non classical (1)	HLA‐DR, CD16, CD11c, CCR7, CD45RA, CD127	CXCR3, Fas_smere_, NKG2c_smere_
84	Monocytes	Non classical (2)	HLA‐DR, CD4, CD16, CD11c, CCR7, CD127, CD141	CXCR3, Fas, NKG2c
85	Monocytes	Non classical (3)	HLA‐DR, CD4, CD16, CD11c, CCR7, CD45RA, CD127, CD141	CXCR3, PD‐1, Fas, NKG2c
86	Myeloid APC	Classical monocytes and CD1c‐CD141‐cDC	HLA‐DR, CD4, CD14^+/−^, CD16, CD11c, CCR7, CD39, CD141, CD25, CD38, CD1c_smere_	CXCR3, PD‐1, CCR5_smere_, Fas, NKG2c
87	Myeloid APC	Non classical monocytes and CD1c^−^CD141^−^ cDC	HLA‐DR, CD4, CD16^+/−^, CD11c, CCR7, CD45RA, CD127, CD141, CD2, CD25, CD27	CXCR3, CCR6_smere_, Fas, NKG2c, NKp30_smere_
88	Myeloid APC	pDC	HLA‐DR, CD123, CD16_smere_, CCR7, CD45RA, CD127, CD141_smere_	CXCR3, NKG2c^+/−^
89	Myeloid APC	pDC and myeloid cells	CD56, HLA‐DR, CD123, CD4, CD14^+/−^, CD16^+/−^, CD11c, CCR7, CD45RA, CD39, IgM, CD25, CD27, CD28	CXCR3, CCR6, PD‐1, CXCR5, NKG2c^+/−^, NKG2D
90	NK	(1)	CD56_smere_, CD16, CCR7, CD45RA, CD127, CD38	CXCR3, NKG2a_smere_
91	NK	(2)	CD56, CD16, CD45RA, CD127, CD38	CXCR3, CD57, NKG2a
92	NK	Early and mature (1)	CD56, CD16^+/−^, CD45RA, CD127, CD38	NKG2a^+/−^, NKG2D, NKp30_smere_
93	NK	Early and mature (2)	CD56, CD16^+/−^, CCR7, CD45RA, CD127, CD38	CXCR3, PD‐1, NKG2a^+/−^, NKG2D, NKp30_smere_
94	NK	Early (1)	CD56, CD16_smere_, CD45RA, CD127	CXCR3, PD‐1, CD57_smere_, Fas_smere_, NKG2a^+/−^, NKG2c, NKG2D
95	NK	Early (2)	CD56, CD16_smere_, CD45RA, CD127, CD38	CXCR3, PD‐1, NKG2a^+/−^, NKG2c, NKG2D, NKp30_smere_
96	NK	Early (3)	CD56, CD45RA	CXCR3, PD‐1, CD57, Fas_smere_, NKG2a^+/−^, NKG2D
97	NK	Early (4)	CD56, CD16_smere_, CD45RA, CD127, CD2	CXCR3, Fas_smere_, NKG2c
98	NK	Early to mature	CD56, CD16, CD45RA	CXCR3, PD‐1, Fas_smere_, CD57, NKG2a^+/−^, NKG2D
99	NK	Mature (1)	CD56, CD16, CD45RA, CD2	CXCR3^+/−^, PD‐1, Fas, C57^+/−^, NKG2a^+/−^, NKG2c, NKG2D
100	NK	Mature (2)	CD56, CD16, CD45RA, CD38	CXCR3^+/−^, PD‐1, NKG2a^+/−^, NKG2c, NKG2D, NKp30_smere_
101	NK	Mature (3)	CD56, CD16, CCR7, CD45RA, CD127, CD38	CXCR3, NKG2a^+/−^, NKG2c, NKG2D
102	NK	Terminal and mature	CD56^+/−^, CD16, CD45RA, CD38	PD‐1, CD57, Fas, NKG2D
103	NK	Terminal (1)	CD16^+/−^, CCR7	CD24, CXCR3, Fas_smere_, NKp30_smere_
104	NK	Terminal (2)	CD16, CD25	CD24, CXCR3_smere_
105	NKT	CD2^−^	CD3, CD56^+/−^, CD8, CD16, CD45RA, CD2_smere_	CXCR3, PD‐1, CD57_smere_, Fas_smere_, NKG2a^+/−^, NKG2c, NKG2D
106	NKT	CD2^+^CD8^−^CD4^+^ (1)	CD3, CD56^+/−^, CD4, CD16, CD2	CXCR3, PD‐1^+/−^, CCR5_smere_, CD57^+/−^, Fas, NKG2D_smere_
107	NKT	CD2^+^CD8^−^CD4^+^ (2)	CD3, CD56^+/−^, CD4, CD127, CD2	CXCR3, PD‐1_smere_, CD57_smere_, Fas_smere_, NKG2c_smere_
108	NKT	CD2^+^CD8^dim^ (1)	CD3, CD56_smere_, CD123, CD4, CD8, CCR7, CD45RA, CD39, IgM, CD127, CD2, CD25, CD28	CXCR3, CCR6, PD‐1, Fas, NKG2D
109	NKT	CD2^+^CD8^dim^ (2)	CD3, CD56_smere_, CD123, CD4, CD8_smere_, CD16, CCR7, CD45RA, CD39, IgM, CD127, CD2, CD25, CD27, CD28	CXCR3, CCR6, PD‐1, CXCR5, Fas, NKG2D
110	NKT	CD2^+^CD8^dim^ (3)	CD3, CD56^+/−^, CD8^+/−^, CD45RA, CD127, CD2	CXCR3, CD57_smere_, Fas_smere_, NKG2c
111	NKT	CD2^+^CD8^high^	CD3, CD56, CD123, CD8, CD16, CCR7, CD45RA, CD39, IgM, CD2, CD25, CD28	CXCR3, CCR6, PD‐1, CCR5, CXCR5, Fas, NKG2D
112	NKT	CD2^−^CD8^−^ and CD2^+^CD8^−^	CD3, CD56, CD16, CCR7, CD45RA, CD127	CXCR3, PD‐1, CCR5_smere_, NKG2a^+/−^, NKG2D
113	NKT	CD2^−^CD8^+^	CD3, CD56, CD8, CD16, CCR7, CD45RA, CD127	CXCR3, CCR5
114	No NK No ILC	(1)	CD16, CCR7_smere_	CXCR3, Fas_smere_, NKG2c_smere_
115	No T no B	(1)	HLA‐DR, CCR7, CD45RA, CD127, CD27	CXCR3, CXCR5, NKG2c_smere_
116	No T no B	(2)	HLA‐DR, CD16, CCR7, CD45RA, CD39, CD127, CD27, CD38	CXCR3, PD‐1, Fas, NKG2c^+/−^
117	No T no B	(3)	HLA‐DR, CCR7, CD45RA, CD39, CD127, CD2_smere_, CD27, CD38	CXCR3, CXCR5, Fas, NKG2c
118	Unknown	CD3^−^TCRγδ^+^ (1)	TCRγδ, CD56, CD123, CD4_smere_, CD16_smere_, CCR7, CD39^+/−^, IgG^+/−^, CD127^+/−^, CD27^+/−^, CD28^+/−^, CD1c_smere_	CXCR3_smere_, CCR6, CCR5^+/−^, CXCR5, Fas_smere_, NKG2c^+/−^, NKG2D^+/−^, NKp30
119	Unknown	CD3^−^TCRγδ^+^ (2)	TCRγδ, CD4_smere_, CD14, CD16^+/−^, CD11c, CCR7, CD127, CD25_smere_, CD27, CD38	CD24, CXCR3, PD‐1, CXCR5_smere_, Fas, NKG2c^+/−^, NKp30
120	Unknown	CD3^−^TCRγδ^+^ (3)	TCRγδ, CD4_smere_, CD14, CD16^+/−^, CD11c, CCR7, CD127, CD141_smere_, CD27, CD38, CD1c	CD24, CXCR3, CXCR5_smere_, Fas, NKG2c, NKp30

### 4.2 Circulating NKT‐Like and Terminal NK Cells Discriminate Between Mild and Severe AP

**FIGURE 2:**
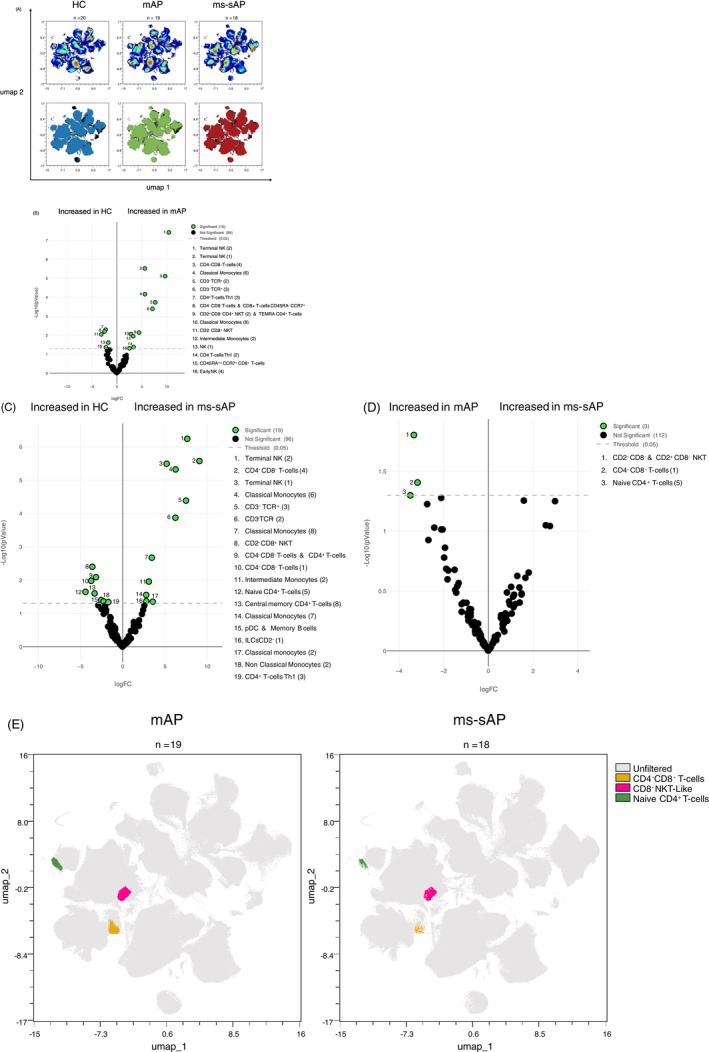
Immunome differences among cohorts. (A) The whole general UMAP plot with all samples was coloured in black, while each cohort is represented in different colours: Blue [healthy control (HC)], Green [mild acute pancreatitis (mAP)], and red [moderate–severe and severe acute pancreatitis (ms‐sAP)], to display the cohort distribution through obtained UMAP. (B) Volcano plots were further used to compare clusters differentially between among HC and mAP; as well as (C) between HC and ms‐sAP; and (D) mAP and ms‐sAP. In green are highlighted those clusters differentially expressed between each comparison (*p*‐value < 0.05). Distribution of the three clusters differentially expressed at hospital admission between patients who subsequently developed mAP or ms‐sAP are shown in (E) where unfiltered refers to whole cells used in unsupervised analysis.

Having described the circulating immunome complexity in our cohorts (Figure 1), we next studied the relative contribution of patients with AP towards this immune complexity (Figure 2A), revealing how patients with AP display, at hospital admission, two specific islands (Figure 2A): one of T‐cells found in the upper side, made of cluster 36 (a subset of CD4^−^CD8^−^ T‐cells) and cluster 120 (a subset of CD3^−^TCRγδ^+^ cells); and another island in the lower side made of cluster 103 (a subset of terminal NK cells) and cluster 119 (another subset of CD3^−^TCRγδ^+^ cells), referred to as controls.

In order to validate these differences, further analysis revealed a total of 16 cellular clusters differentially expressed between controls and patients with subsequent mAP, with the latter displaying a marked increase in terminal NK cells, classical monocytes and non‐classical T‐cells (CD4^−^CD8^−^ as well as CD3^−^TCRγδ^+^) (Figures 2B and S2A). When controls were compared with AP patients with subsequent ms‐sAP, a similar trend was found with a total of 19 clusters differentially expressed among them, with the latter displaying again an expansion of terminal NK cells, classical monocytes and non‐classical T‐cells (CD4^−^CD8^−^ as well as CD3^−^TCRγδ^+^) (Figures 2C and S2B) hence highlighting the similarities between mAP and ms‐sAP.

Having said that, a further analysis was specifically performed comparing the immune profile of patients with AP at hospital admission based on their subsequent clinical evolution. Hence, our results revealed how patients with subsequent mAP had a specific expansion of CD8^−^ NKT‐like cells, CD3^+^TCR^−^CD56^+^ cells, CD4^−^CD8^−^ T‐cells and Naïve CD4^+^ T‐cells referred to those who developed more aggressive forms of the disease (Figure 2D), as also shown in Figure 2E.

### 4.3 A Validated NKT‐Like Signature Predicts Acute Pancreatitis Severity

**FIGURE 3:**
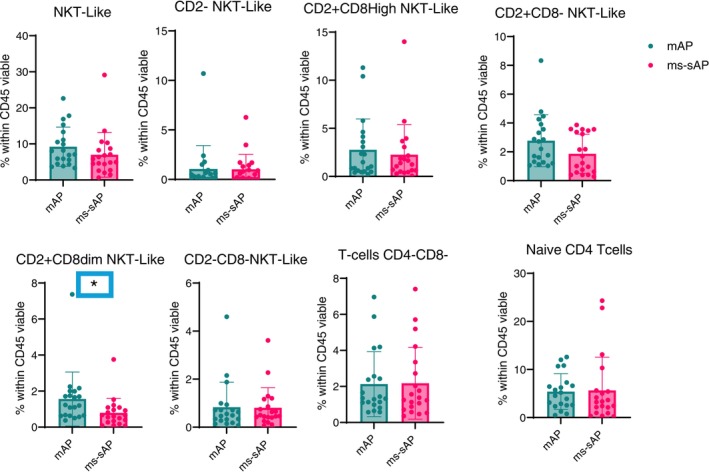
Hierarchical gating validation of the clusters differentially found among mild and moderate severe–severe patients. The cell clusters differentially found at hospital admission between patients with acute pancreatitis who subsequently had mild acute pancreatitis (mAP), or moderate–severe and severe acute pancreatitis (ms‐sAP) were identified by classical gating strategies as shown in Figure 3. There relative proportion in each cohort was subsequently referred to total circulating single viable CD45^+^ cells. Unpaired *t*‐test was applied and *p*‐value < 0.05 was considered significant (* < 0.05).

Having described how 3 cellular clusters can predict AP outcome at hospital admission (Figure 2D), we next aimed to validate these findings by classical or hierarchical cell identification. Hence, and although with our panel a total of 70 different immune cell subsets could be identified (Figure S3), the relative proportion at hospital admission was only determined for those subsets related to these three clusters including total NKT‐Like cells (and their respective subsets), as well as CD4^−^CD8^−^ T‐cells and naïve CD4^+^ T‐cells (Figure 3). Among them, our results confirmed that CD2^+^CD8^dim^ NKT‐like cells were decreased at hospital admission in patients with subsequent ms‐sAP, hence hypothesizing the role of this immune cell subset as a novel biomarker with capacity to support prediction for disease outcome in patients with AP at hospital admission.

### 4.4 Functional Rewiring of NKT‐Like Responses in Ms‐sAP Versus mAP

To further explore the role of NKT‐like cells in AP, not just as a novel biomarker to discern between mAP and ms‐AP, but also as potential mediators of disease progression, their function was further assessed in a prospective cohort.

Hence total PBMC from patients with subsequent mAP and ms‐sAP were stimulated in the presence of IL‐15 (referred to the paired basal culture), and the expression of activation markers (CD69 and CD25), markers of NK‐cell function (NKp30, NKG2a, NKG2D and NKG2c) and their cytotoxic capacity (Granzyme and Perforin) was determined on each NKT‐like cell subset.

**FIGURE 4:**
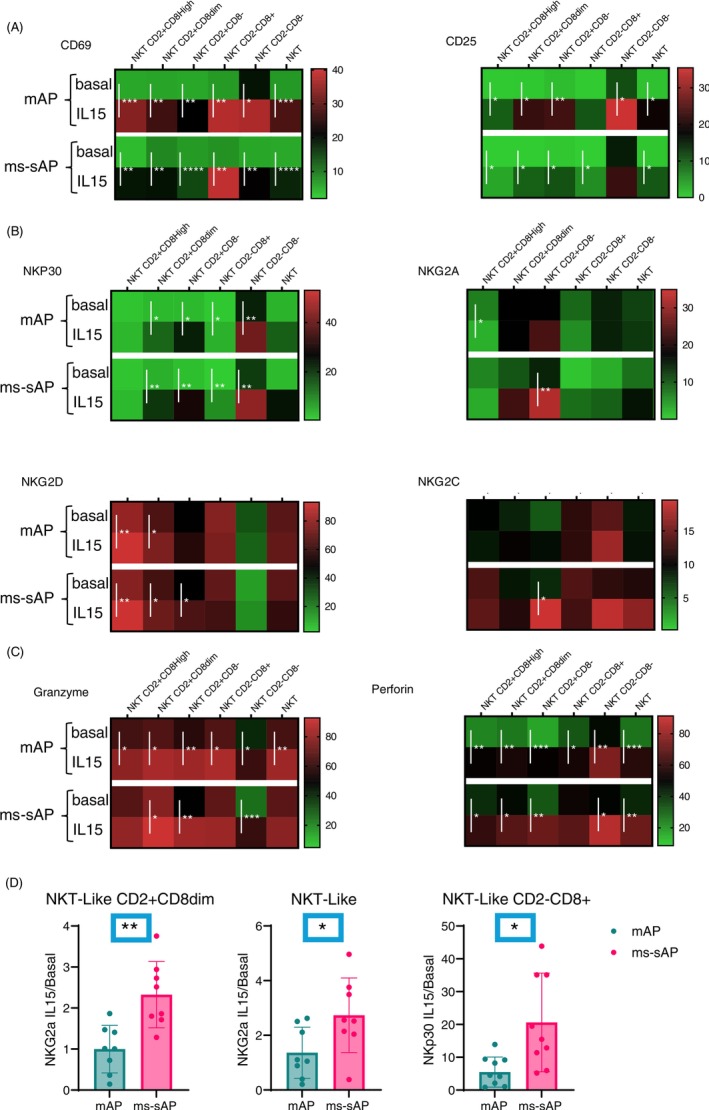
Functional characterization of circulating NKT‐Like cells in patients with acute pancreatitis at hospital admission. Peripheral blood mononuclear cells from patients with mild acute pancreatitis (mAP), or moderate–severe and severe acute pancreatitis (ms‐sAP) were overnight cultured in the presence/absence of IL‐15. Cells were identified as in Figure S4A and the expression level of each marker determined as in Figure S4B. Expression of activation markers CD69 and CD25 for both conditions in each cohort is shown as a heatmap in (A) while the expression of markers of NK‐cell function (NKp30, NKG2a, NKG2D and NKG2c) is displayed in (B), and their cytotoxic capacity (Granzyme and Perforin) in (C). The IL‐15/basal ratio for those markers and NKT‐cell subsets differentially induced between patients with mAP and ms‐AP is shown in (D). Paired *t*‐test was applied in panels (A–C) while unpaired *t*‐test was used in panel (D) in all cases *p*‐value < 0.05 was considered significant (* < 0.05; ** < 0.01; *** < 0.001; **** < 0.0001).

Following IL‐15 stimulation, most NKT‐like subsets were activated (as determined by the expansion of CD25 and CD69), although that was not the case for CD25 on CD2^−^CD8^+^ NKT‐cell subsets from mAP patients (Figure 4A). On the contrary, the expression of NK receptors revealed a heterogenous IL‐15 response among both cohorts (Figure 4B) since these markers were typically more induced in patients with ms‐sAP. Indeed, NKG2A, NKG2C and NKG2D were specifically induced by IL‐15 stimulated CD2^+^CD8^−^ NKT‐like cells from ms‐sAP patients. On the contrary, granzyme and perforin displayed an inverted pattern since they were typically more induced in patients with mAP (Figure 4C) as also shown by the fact that both markers were specifically induced by IL‐15 on CD2^−^CD8^+^ NKT‐like cells from mAP patients, who also had an increase of granzyme on CD2^+^CD8^high^ NKT‐like cells. Having said that, further analysis confirmed that NKG2A expression was more enhanced on patients with ms‐sAP following IL‐15 stimulation on total NKT‐like as well as on CD2^+^CD8^dim^ NKT‐like cells, while NKp30 was further enhanced on CD2^−^CD8^+^ NKT‐like cells (Figure 4D). Together, these results support therefore the role of NKT‐like cells not just as potential biomarkers, but also as immune subsets which could mediate disease progression in ms‐sAP.

## 5 Conclusions

We hereby provide, using an innovative spectral cytometry approach, a comprehensive immune characterization of AP and identify a possible novel immunological biomarker capable of distinguishing between subsequent mAP and ms‐sAP at early stages of hospital admission. Indeed, our results reveal that NKT‐like cells may elicit a central role in AP since patients with subsequent severe outcomes have lower levels of circulating CD2^+^CD8^dim^ NKT‐like cells at hospital admission which, nevertheless, display a higher activation capacity. Together, the decrease of these circulating subsets coupled with their expanded function suggest that CD2^+^CD8^dim^ NKT‐like cells may be mediating some of the mechanisms driving disease progression in ms‐sAP.

From a translational perspective, our findings point to the potential use of NKT‐like cells as biomarkers for early risk stratification in AP. Peripheral immune profiling could therefore serve as a minimally invasive tool for predicting disease severity upon hospital admission. Indeed, this is the first time, to the best of our knowledge, where a specific reduction of circulating NKT‐like cells has been described in AP. Nevertheless, similar observations have already been made in several other inflammatory conditions, particularly in autoimmune diseases. For instance, patients with systemic lupus erythematosus exhibit significantly lower levels of circulating NKT‐like cells compared to HCs [20]. Similarly, Zhou et al. [21] found that reduced NKT‐like cell levels correlated with increased clinical severity in primary Sjögren's syndrome. NKT‐like cell diminution has also been reported in malignant disorders like hepatocellular carcinoma, where such a decrease was associated with reduced overall survival [22] while in patients with chronic lymphocytic leukaemia, their decreased levels were observed in patients with progressive disease [23, 24].

In line with our findings, lymphocyte depletion (particularly CD4^+^ T‐cells) has been associated with severe outcomes in AP [25, 26]. This phenomenon is believed to result from immune cell infiltration into inflamed pancreatic tissue, disrupting peripheral immune homeostasis and contributing to systemic immune dysregulation [27]. In this context, our results support the hypothesis that NKT‐like cells may migrate from blood into the pancreas during severe disease, thereby reducing their systemic immunoregulatory function and contributing to an exaggerated local immune response that exacerbates tissue damage and clinical severity. Indeed, we have also seen that NKT‐like cells from patients with ms‐sAP display a heightened cytotoxic capacity in response to a well‐known activator of the innate immune system such as IL‐15; hence, confirming that the immune system from patients with subsequent ms‐sAP is not only quantitatively altered but also functionally unbalanced towards increased cytotoxicity. Therefore, this proinflammatory profile may play a critical role in amplifying pancreatic injury and systemic inflammation, ultimately driving worse clinical outcomes. Indeed, previous reports have also shown similar cytotoxic NKT‐like phenotypes in chronic myeloid leukaemia, together supporting the relevance of these alterations across disease contexts [28].

In summary, our study reveals that patients with mAP have higher levels of circulating NKT‐like cells with lower cytotoxic activity, whereas patients with severe disease display a reduction in these cells and a stronger cytotoxic response upon stimulation. These features may therefore contribute to disease worsening through local immune overactivation and systemic immune imbalance. As a consequence, modulating NKT‐like cell function may represent a novel therapeutic avenue for controlling excessive inflammation and preventing progression to organ failure. Further studies in independent cohorts are therefore warranted to validate these observations and to explore their clinical applicability in larger, prospective cohorts.

## Author Contributions

**Carolina González de Castro:** laboratory experiments and sample processing, spectral cytometry experiments, data analysis and interpretation, drafting of the manuscript, cytometry data preprocessing and quality control. **Mª. Lourdes Ruiz Rebollo:** conception and design of the study, study design and supervision. Obtention of the funding. **Paloma Cal‐Sabater:** laboratory experiments and sample processing, cytometry data preprocessing and quality control. **Elisa Arribas‐Rodríguez:** laboratory experiments and sample processing. **Álvaro Martín‐Muñoz:** spectral cytometry experiments, cytometry data preprocessing and quality control. **Alejandro Gonzalez del Hierro:** laboratory experiments and sample processing. **Marina Perez Mazzali:** laboratory experiments and sample processing. **Jessica Matesanz‐Isabel:** laboratory experiments and sample processing. **Hugo Gonzalo‐Benito:** conception and design of the study, study coordination and project administration. **Sara Cuesta‐Sancho:** conception and design of the study, study design and supervision, data analysis and interpretation, drafting of the manuscript, Study coordination and project administration. **David Bernardo:** conception and design of the study, study design and supervision, study coordination and project administration, obtention of the funding.

## Funding

This work was funded by the Spanish Ministry of Science (PID2023‐148270OB‐I00) and Junta de Castilla y León (IR2020‐1‐UVA01, IR2021‐UVA04).

## Ethics Statement

The study was performed in accordance with the declaration of Helsinki and it was approved by the Research and Ethical Board of our institution (CEIC PI‐GR‐22‐2825).

## Consent

Participants (patients and healthy controls) provided signed informed consent to be included in the study. All data were coded.

## Conflicts of Interest

The authors declare no conflicts of interest.

## Supporting information


**Table S1:** Antibody panel for the discovery cohort.
**Table S2:** Antibody panel for the validation cohort.
**Figure S1:** Cluster heatmap.
**Figure S2:** Clusters significantly different between different cohorts.
**Figure S3:** Hierarchical cell subsets identification.
**Figure S4:** Gating strategy on the validation cohort.

## Data Availability

The data that support the findings of this study are available from the corresponding author upon reasonable request.
