# Supplementary material for: High‐Dimensional Immune Profiling Identifies Circulating NKT‐Like Cells Associated With Severity Outcome in Acute Pancreatitis at Disease Onset
Source: Immunology. 2026 May 12;178(4):685–703. doi: 10.1111/imm.70143 (PMC13327138; doi:10.1111/imm.70143)

**SUPPLEMENTARY TABLES**

**Supplementary table 1. Antibody panel for the discovery cohort.**

Antibody specificity, fluorochrome, clone and manufacturer for each of the antibodies used. Each sample has been stained with all 40 antibodies displayed on this table. Fluorochrome, marker, clone and supplier are the main identifier labels shown here.

| **Specificity** | **Fluorochrome** | **Clone** | **Manufacturer** |
| --- | --- | --- | --- |
| CCR5 (CD195) | BUV 563 | 2D7/CCR5 | BD |
| CCR6 (CD196) | BV 711 | G034E3 | BioLegend |
| CCR7 (CD197) | BV 421 | G043H7 | BioLegend |
| CD11c | eFluor 450 | 3,9 | eBioscience |
| CD123 | Super Bright 436 | 6H6 | eBioscience |
| CD127 | APC/R700 | HIL-7R-M21 | BD |
| CD14 | Spark Blue 550 | 63D3 | BioLegend |
| CD141 | BB 515 | 1A4 | BD |
| CD16 | BUV 496 | 3G8 | BD |
| CD19 | Spark NIR 685 | HIB19 | BioLegend |
| CD1c | Alexa Fluor 647 | L161 | BioLegend |
| CD2 | PerCP/Cy5.5 | TS1/8 | BioLegend |
| CD20 | Pacific Orange | HI47 | Invitrogen |
| CD24 | PE/Alexa Fluor 610 | SN3 | Thermo Fisher |
| CD25 | PE/Alexa Fluor 700 | 3G10 | Thermo Fisher |
| CD27 | APC/H7 | M-T271 | BD |
| CD28 | BV 650 | CD28.2 | BioLegend |
| CD3 | BV 510 | SK7 | BioLegend |
| CD38 | APC/Fire 810 | HB-7 | BioLegend |
| CD39 | BUV 661 | TU66 | BD |
| CD4 | cFluor YG584 | SK3 | Cytek |
| CD45 | PerCP | 2D1 | BioLegend |
| CD45RA | BUV 395 | 5H9 | BD |
| CD56 | BUV 737 | NCAM16.2 | BD |
| CD57 | FITC | HNK-1 | BioLegend |
| CD8 | BUV 805 | SK1 | BD |
| CD95 (Fas) | PE/Cy5 | DX2 | BioLegend |
| CXCR3 (CD183) | PE/Cy7 | G025H7 | BioLegend |
| CXCR5 | BV 750 | RF8B2 | BD |
| HLA-DR | PE/Fire 810 | L243 | BioLegend |
| IgD | BV 480 | IA6-2 | BD |
| IgG | BV 605 | G18-145 | BD |
| IgM | BV 570 | MHM-88 | BioLegend |
| NKG2A (CD159a) | APC | REA110 | Miltenyi |
| NKG2C (CD159c) | PE | REA205 | Miltenyi |
| NKG2D (CD314) | BUV 615 | 1D11 | BD |
| NKp30 (CD337) | PE/Dazzle 594 | P30-15 | BioLegend |
| PD-1 (CD279) | BV 785 | Eh12.2H7 | BioLegend |
| TCRyg | PerCP/eFluor 710 | B1.1 | eBioscience |

**Supplementary table 2. Antibody panel for the validation cohort..** Each sample has been stained with all 17 antibodies displayed on this table. Fluorochrome, marker, clone and supplier are the main identifier labels shown here.

| **Specificity** | **Fluorochrome** | **Clone** | **Manufacturer** |
| --- | --- | --- | --- |
| CD2 | PerCP/Cy5.5 | TS1/8 | BioLegend |
| CD25 | PE/Alexa Fluor 700 | 3G10 | Thermo Fisher |
| CD3 | BV 510 | SK7 | BioLegend |
| CD4 | cFluor YG584 | SK3 | Cytek |
| CD45 | PerCP | 2D1 | BioLegend |
| CD56 | BUV 737 | NCAM16.2 | BD |
| CD69 | PE/Cy7 | FN50 | BioLegend |
| CD8 | BUV 805 | SK1 | BD |
| Granzyme B | FITC | QA16A02 | BioLegend |
| NKG2A (CD159a) | APC | REA110 | Miltenyi |
| NKG2C (CD159c) | PE | REA205 | Miltenyi |
| NKG2D (CD314) | BUV 615 | 1D11 | BD |
| NKp30 (CD337) | PE/Dazzle 594 | P30-15 | BioLegend |
| Perforin | BV421 | dG9 | BioLegend |
| TCRyg | PerCP/eFluor 710 | B1.1 | eBioscience |
| Viability | Live Dead UV Blue | - | Thermo Fisher |

**SUPPLEMENTARY FIGURES**

**Supplementary Figure 1. Cluster heatmap.**

The heatmap displays the intensity levels for each marker on each of the 120 clusters identified by FlowSOM. A colour code was based on the expression intensity, where red represent higher expression and the transition to blue represent lower expression.

**
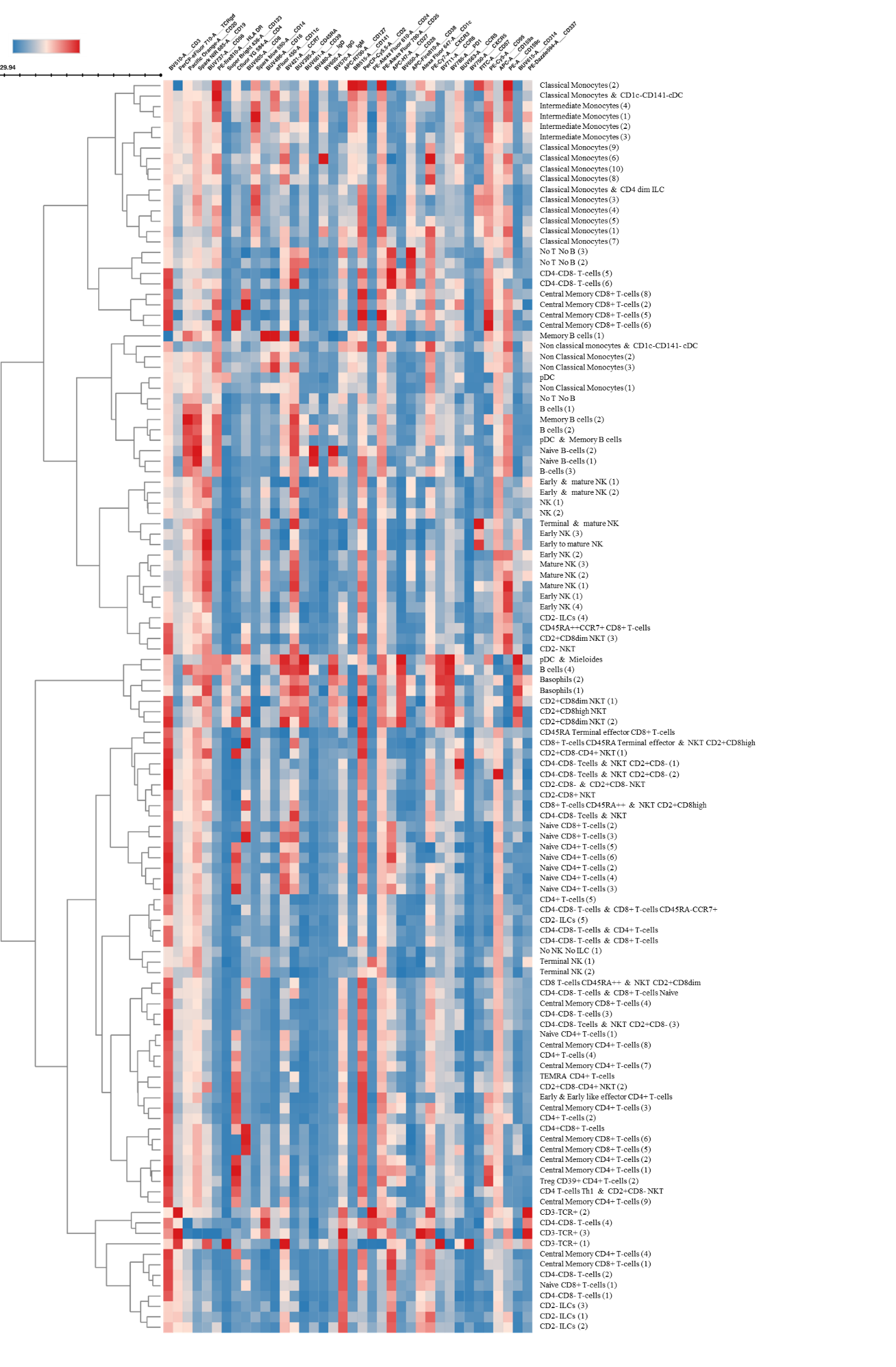
**

**Supplementary Figure 2. Clusters significantly different between different cohorts.**

Clusters differentially expressed at hospital admission between Healthy controls (HC) and patients with mild acute pancreatitis (mAP) are shown in A) where unfiltered refers to whole cells used in unsupervised analysis. Among mAP and moderate severe-severe (ms-sAP) patients there are 19 clusters differentially expressed, and those are shown in B).

**
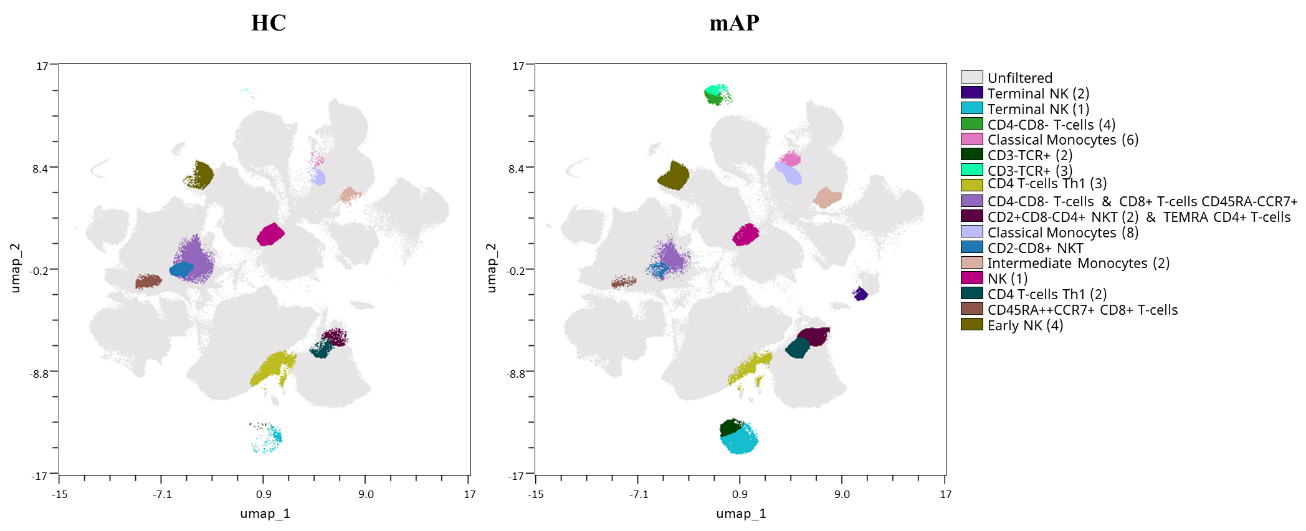
**

**
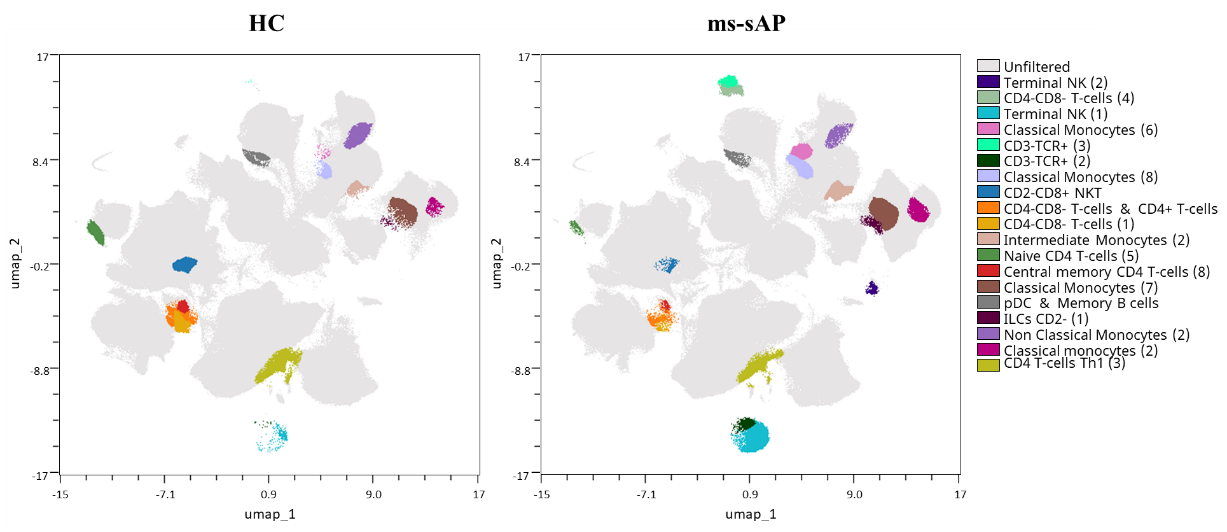
**

**Supplementary Figure 3. Hierarchical cell subsets identification.**

A total of 70 different immune cell subsets were identified within total singlet viable peripheral blood mononuclear cells (PBMCs) by hierarchical a gating approach. Arrows indicate how different plots are interwind with the rest. Starting by exclusion of doublets and dead cells, Total leukocytes are identified based on the expression of CD45. Tγδ [1] cells are delineated as CD45^+^CD3^+^TCRγδ^+^ and further subdivided attending CD45RA and CCR7. Within the CD45^+^CD3^-^TCRγδ^-^ fraction, two main groups can be separated: No T no B cells are defined as CD19^-^CD20^-^ fraction while B-cells are the remaining. Within the No T no B fraction, attending to HLA-DR expression, Basophils [2] are HLA-DR^-^CD123^+^CD38^+^, meanwhile NK cells [3] were identified as CD3-TCRγδ^-^CD19^-^CD20^-^HLA-DR^-^ and classified as Early NK (CD56^+^CD16^-^), mature NK (CD56^+^CD16^+^) and terminal NK (CD56^-^CD16^+^). Innate lymphoid cells (ILCs) [4] are defined as double negative fraction CD16^-^CD56^-^CD127^+^and further divided based on CD2 expression. CD2^+^ cells within can further separated into subsets based on the expression of CD4 and CD8. Among No T no B cells HLA-DR^+^ fraction, based on CD11c expression it can be differentiated Myeloid APC [5] or Plasmacytoid dendritic cells (pDC) [6], which are further defined as CD123^+^. Myeloid are defined as HLA-DR^+^CD11c^+^. Within them Non classical monocytes are CD14^-^CD16^+^, Intermediate monocytes CD14^+^CD16^+^ and classical monocytes CD14^+^CD16^-^, while double negative CD14^-^CD16^-^ cells are defined as Total classical dendritic cells (cDC). The latter were further divided into type 1 (CD1c^-^CD141^+^), type 2 (CD1c^+^) and cDC1^-^CD141^-^ cDC. B cells [7] were gated out of CD3^-^TCRγδ^-^ as CD19^+^ and/or CD20^+^, and further gated as IgD^+^CD27^-^, IgD^+^CD27^+^, or IgD^-^CD27^+/-^. The IgD-CD27^+/-^ subset was divided into plasmablasts or IgD^-^ memory B cells based on CD20 expression and CD27, and IgM or IgG expression was measured in each of them. As for the CD3^+^TCRγδ^-^fraction, .CD56 was used to identify total NKT-like cells as CD3^+^TCRγδ^-^CD56^+^ [8], which were subdivided attending CD2 and CD8 expression. Last, total T-cells were defined as CD3^+^TCRγδ^-^CD56^-^ [9] that were further divided into CD4^+^, CD8^+^, CD4^+^CD8^+^ and CD4^-^CD8^-^ T-cells. Regulatory T-cells were identified within total CD4^+^ T-cells as CD127^-^CD25^+^. CD39 and CD45RA were used to further divide them. Within total CD4^+^ T-cells or CD8^+^ T-cells, CCR7 and CD45RA, were used to identify total naïve, central memory, effector memory and TEMRA, while CD27 and CD28 were further used to define early, early-like and terminal effector T-cells within the effector memory fraction. In a similar manner, within central memory CD4^+^ T-cells was further used to identify Tfh cells. By last using CXCR3 and CCR6 expression Th1, Th2 and Th17 cells could be identified among every effector CD4 subset as well as among central memory CD4.

**
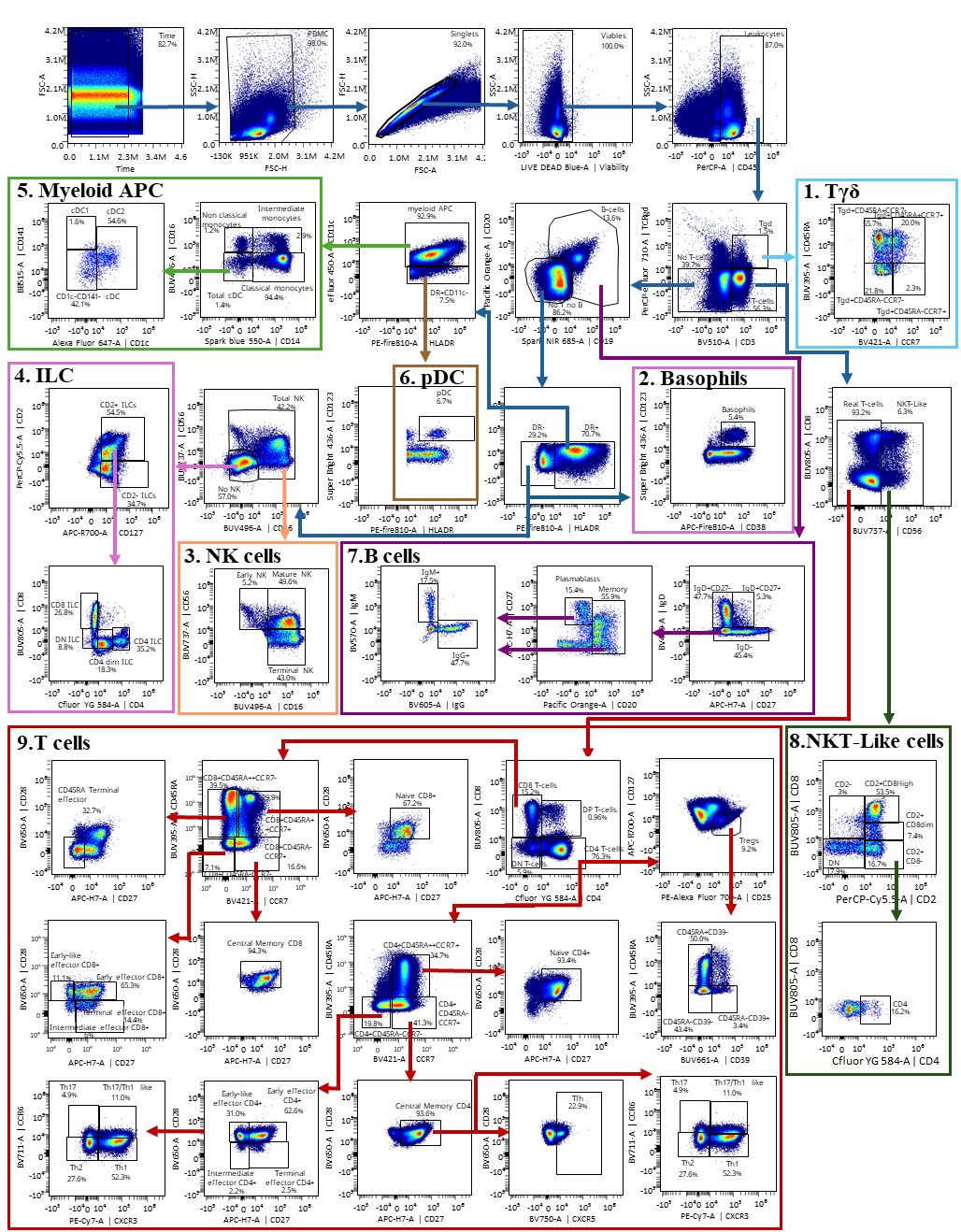
**

**Supplementary Figure 4. Gating strategy on the validation cohort.**

Analysis of functional validation cohort, cohort 2, stained with a reduced panel, shown in **Supplementary Table 2**, allowed this hierarchical gating strategy. A total of 10 different immune cell subsets were able to be identified within total singlet viable peripheral blood mononuclear cells (PBMCs), shown in panel **A)**. Arrows indicate how different plots are interwind with the rest. Starting by exclusion of doublets and dead cells, total viable leukocytes are identified based on the expression of CD45^+^ and viability^-^. Within them, CD3 expression divides cells on one hand as No T cells, among which only NK cells are identified as CD56^+^ fraction, and on the other hand as total T cells. Based on CD56 expression it can be differentiated as CD56^-^ T cells, subsets based on CD4 and CD8 expression, and as NKT-Like cells which are CD56^+^ fraction. Using CD2 and CD8, five NKT-Like subsets are obtained: CD2^-^CD8^-^, CD2^-^CD8^+^, CD2^+^CD8^High^, CD2^+^CD8^dim^ and CD2^+^CD8^-^. Functional markers included in the panel were evaluated following its expression among each of subsets of interest. An example of its measurement is shown in **B)**, where CD69 expression is shown, both in resting conditions and after IL-15 culture, on both total and CD2^+^CD8^dim^ NKT-like cells.


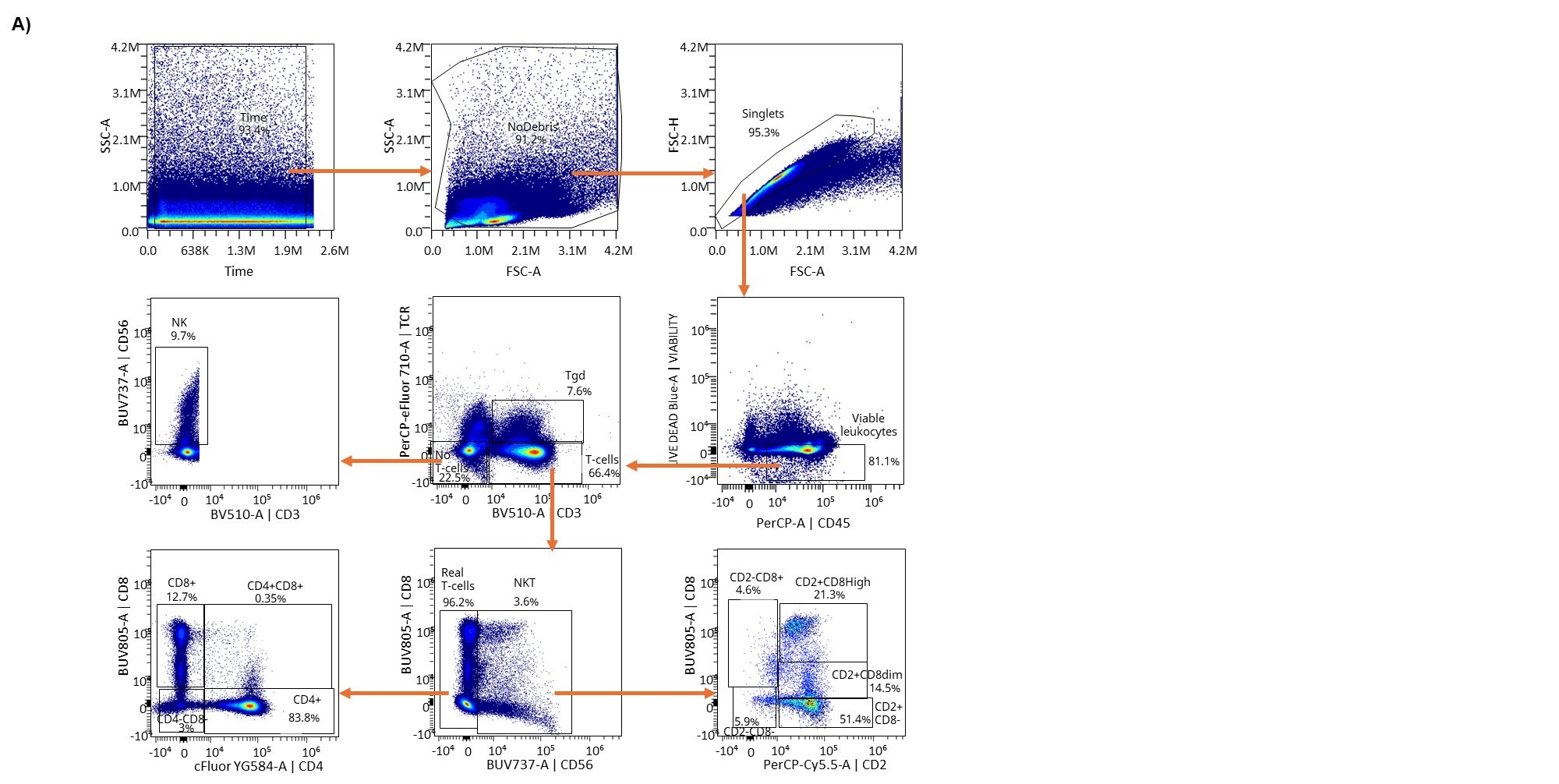


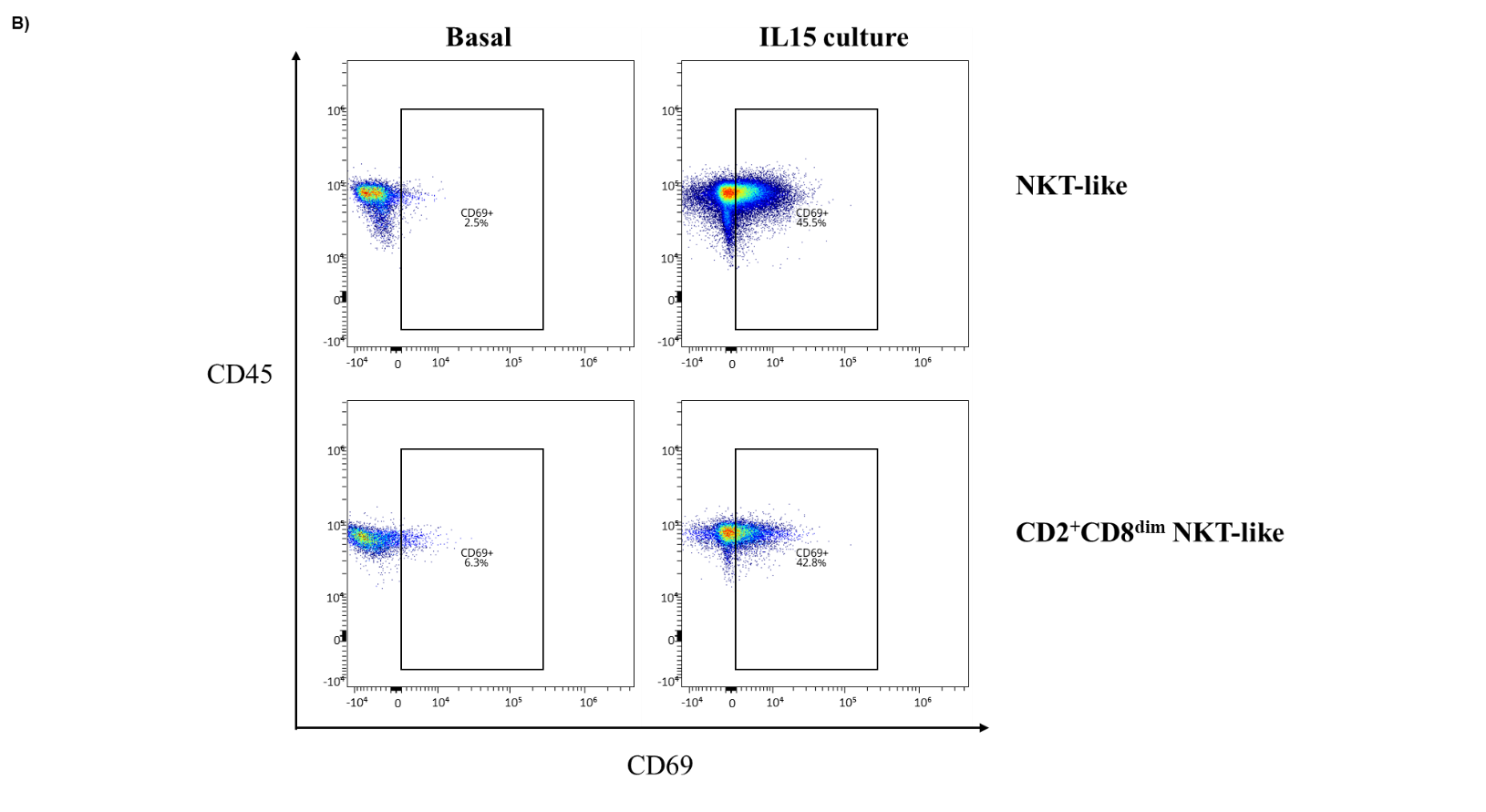

Supplement: Supplementary file 1 — Table S1: Antibody panel for the discovery cohort. Table S2: Antibody panel for the validation cohort. Figure S1: Cluster heatmap. Figure S2: Clusters significantly different between different cohorts. Figure S3: Hierarchical cell subsets identification. Figure S4: Gating strategy on the validation cohort. [file IMM-178-685-s001.docx]
